# Penicillin: from discovery to patenting the large-scale production process

**DOI:** 10.1590/S0104-59702024000100021

**Published:** 2024-05-17

**Authors:** Roberto Viana da Silva, Rita Pinheiro-Machado, Ana Cristina Augusto de Sousa

**Affiliations:** i Analista de gestão, Fundação Oswaldo Cruz. Rio de Janeiro – RJ – Brasil roberto.viana@fiocruz.br; ii Especialista sênior, Instituto Nacional da Propriedade Industrial. Rio de Janeiro – RJ – Brasil ritap@inpi.gov.br; iii Pesquisadora em saúde pública, Fundação Oswaldo Cruz. Rio de Janeiro – RJ – Brasil anacris.sousa@ensp.fiocruz.br

**Keywords:** National security, Industrial property, Innovation, Segurança nacional, Propriedade industrial, Inovação

## Abstract

This article examines discoveries, inventions, and innovations related to penicillin by sampling activities to solve technological problems which can be traced by the distribution of scientific articles, government reports, innovations, and patents between 1929 and 1945, and proposes reflection on the importance of scientific progress for national security. The analysis highlights the technological trajectory and outcomes in the area of intellectual property, considering US policy implemented to catalyze innovation and provide institutional conditions to meet national defense needs as an important factor, although this did not necessarily imply a unique solution in other contexts.

## Resumo

A partir de pesquisa sobre a descoberta, a invenção e a inovação relacionadas à penicilina, por amostra de atividades de resolução de problemas tecnológicos rastreada pela distribuição, no período de 1929 a 1945, de trabalhos científicos, relatórios de governo, inovações e patentes, o artigo propõe uma reflexão sobre a importância do progresso científico para a segurança nacional. A análise destaca a trajetória tecnológica e os resultados na área de propriedade intelectual, considerando um fator importante a política implementada nos EUA para catalisar processos de inovação e oferecer condições institucionais para atender às demandas de defesa nacional, o que não significa necessariamente unicidade de solução em outros contextos.

ARTIGO_Roberto_Rita_ACristina.indd 1 19/04/2024 14:12:08

Durante milhares de anos de desenvolvimento cognitivo – característica evolutiva mais significativa do *Homo sapiens* –, em ambientes severos e de intensa disputa biológica, cujo objetivo primordial era sobreviver, gradualmente foram criados meios para mais entendimento sobre a natureza e a satisfação das necessidades humanas. Essa dinâmica, caracterizada pela organização e aplicação de processos mentais, criou uma base de conhecimento para a solução de problemas técnicos, além dos limites da descoberta de fenômenos e leis naturais (Dalgalarrondo, 2009).

Com as técnicas primitivas, o ser humano já demonstrava o seu caráter inventivo e a capacidade de lidar com as adversidades, como, por exemplo, criando objetos de defesa, por meio do controle e uso do fogo na forja de metais (Lucrécio, 1962). Desde então, a "prontidão do homem para resolver as diferenças pela força das armas tem sido uma característica da sociedade" (Swing, 1991, p.vii).[1] Esse, aliás, é um dos motivos por que na contemporaneidade os textos sobre segurança destacam a natureza contínua dessa relação, sendo difícil não abordar os seus efeitos sem mencionar as mortes violentas causadas, por se tratar do impacto mais direto e tangível.

Quanto aos inimigos invisíveis (a olho nu), os micro-organismos patogênicos, esses seres se disseminaram pelo mundo há muito tempo não somente como ameaças biológicas deliberadas ou acidentais, mas previamente como ocorrências naturais, impactando a história e a cultura humanas (McNeill, 1976). Embora eles tenham exigido um esforço intelectual do ser humano desde a Antiguidade, remontam ao século XVII, com a microscopia, e posteriormente ao século XIX, com a microbiologia e os estudos relacionados à epidemiologia, os meios científicos de comprovar a existência e compreender as formas de transmissão de doenças infecciosas[2] (Roitman, 16 maio 2009).

Ante as várias fontes de ameaças existentes e medidas de defesa que podem ser implementadas em diferentes níveis de generalidade e especificidade, alguns países têm se mobilizado para lidar com o grau de insegurança por elas provocado, geralmente medido em relação às vulnerabilidades com "potencial de derrubar ou enfraquecer significativamente as estruturas do Estado, tanto territoriais como institucionais, assim como o regime político" (Ayoob, 1991, p.259). Diante desse processo, é justificável a adoção de estratégias nacionais com escopo mais amplo, além da dimensão militar, que inclua também a esfera civil e considere todo espectro de "políticas, estruturas, métodos, planos e procedimentos envolvidos na manutenção ou restauração da biossegurança após uma ocorrência natural, acidental ou deliberada de micro-organismos patogênicos e toxinas" (Bonin, 2007, p.28).

A relação entre a essencialidade do progresso científico e a segurança nacional, uma das justificativas utilizadas no relatório "Science, the endless frontier" (Bush, 1945) para descrever a mobilização da ciência, tecnologia e inovação nos EUA durante a Segunda Guerra Mundial, como, por exemplo, em relação à pesquisa e ao desenvolvimento tecnológico de um processo de produção em larga escala de penicilina, recentemente persuadiu estudiosos e formuladores de políticas a invocar a ciência como ponto vital no enfrentamento de fenômenos pandêmicos contemporâneos (Azoulay, Jones, 2020; Best, Bradley, 2020; Gross, Sampat, 2020; Lindee, 1 abr. 2020; Markey, Slavin, 17 mar. 2020; Navarro, 2020), assim como a reconhecer as doenças infecciosas como uma ameaça ao funcionamento do Estado e da sociedade (Kamradt-Scott, McInnes, 2012).

ARTIGO_Roberto_Rita_ACristina.indd 2 19/04/2024 14:12:08

Agindo para que aspectos complexos de invenção e inovação não fiquem situados à margem da história da penicilina, geralmente exposta em termos da sua descoberta, este artigo busca descrever a sua trajetória tecnológica,[3] que atravessa um período da indústria farmacêutica "em que havia pouco desenvolvimento de novos medicamentos e no qual a pesquisa mínima conduzida era baseada em métodos relativamente primitivos" (Henderson, Orsenigo, Pisano, 1999, p.269). A análise leva em consideração os dados e as informações dos principais elementos da descoberta, da invenção e da inovação, assim como os resultados na área de propriedade intelectual, seguindo uma abordagem qualitativa que utiliza pesquisa bibliográfica e documental, por amostra de atividades de resolução de problemas tecnológicos (processo e produto), rastreada pela distribuição no período de 1929 a 1945 de trabalhos científicos, relatórios de governo, inovações e patentes relacionados à penicilina.

## Breves antecedentes históricos e aspectos contemporâneos

Não é desarrazoado considerar que, em determinados momentos da história, os principais adversários nas guerras não eram os soldados inimigos, mas os micro-organismos que causavam doenças patogênicas. É o que se depreende do relatório produzido e compartilhado por Florence Nightingale (1858) com lorde Panmure, secretário de Estado da Guerra do Reino Unido, sobre as questões que afetaram a saúde, a eficiência e a administração hospitalar do Exército britânico, principalmente relacionadas às deficiências logísticas e médicas na Guerra da Crimeia (1853-1856). No caso, das 18.058 mortes reportadas no período de abril de 1854 a junho de 1856, apenas 2.314 foram classificadas como mortes violentas, representando as por doenças infecciosas 14.507, ou seja, um percentual de 80,33%.[4]

No século XX, a malária[5] foi um adversário inesperado em vários cenários da Primeira Guerra Mundial, em que a circulação de vetores e os reservatórios humanos de infecção favoreceram a evolução do parasita causador, deixando pelo menos 1,5 milhão de soldados infectados e um índice de mortalidade de 0,2 a 5% (Brabin, 2014). Com o mundo ainda sob os efeitos do conflito e o número de homens mortos estimado em nove milhões (Faron, 2002), uma mutação do vírus *influenza* foi a causa de uma pandemia, denominada gripe espanhola, determinante para um novo aumento da taxa de mortalidade no mundo (WHO, 2020).

Após desempenhar um papel relativamente importante na Primeira Guerra Mundial, a ciência e a tecnologia foram protagonistas na Segunda Guerra Mundial – como resultado das descobertas, invenções e inovações, doenças naturais e ferimentos relacionados ou não com as batalhas passaram a ser tratadas com antibióticos,[6] vacinas, medidas de higiene e novos métodos, os quais trouxeram avanços surpreendentes, reduzindo radicalmente a incidência de mortalidade e de outras infecções causadas por micro-organismos patogênicos (Bush, 1945; Acemoglu, Johnson, 2007).

Na dimensão política e estratégica, o pós-guerra apresentou ações mais contundentes, de forma a não restringir o tema segurança aos limites estreitos de ameaças militares ou concentrado no Estado e no conflito, sendo expandido para incluir áreas como saúde. Em 1948, por exemplo, o Congresso norte-americano reconheceu que as doenças da humanidade, por causa de sua abrangência, seus efeitos e impactos na saúde humana, constituíam-se em óbice ao desenvolvimento de vários países e declarou que uma das políticas dos EUA nas relações

ARTIGO_Roberto_Rita_ACristina.indd 3 19/04/2024 14:12:08

internacionais era manter e fortalecer os esforços mútuos entre as nações nas pesquisas contra várias doenças (EUA, 1948). Na década de 1950, foi estabelecida uma cooperação entre a Sociedade Geográfica Americana de Nova York, as Forças Armadas dos EUA e as corporações farmacêuticas internacionais, no âmbito de um programa de geografia médica, cuja principal iniciativa visava à elaboração de um atlas para documentar a disseminação global de várias doenças[7] (Acemoglu, Johnson, 2007; Barney, 2014).

Do mesmo modo, estavam em andamento estudos estratégicos na área de propriedade intelectual, em especial sobre a importância do sistema de patentes, e como o poder estatal poderia ser fortalecido (Bush, 1945). Com o fim de formular uma política uniforme e sólida, foram publicados, em 1947, os resultados de uma investigação de práticas e políticas governamentais nessa área conduzida pelo Departamento de Justiça dos EUA. O estudo comparado das leis de vários países quanto ao controle dos direitos de patentes resultantes de pesquisa governamental desenvolvida por servidores e contratados, especialmente aqueles cujos sistemas de patentes e desenvolvimento industrial se aproximavam da realidade americana, demonstrou que, em maior ou menor grau, via legal ou contratual, vários países já regulavam a matéria (EUA, 1947).

Com a evolução dos estudos estratégicos na área de segurança, já no período pós-Guerra Fria, é possível observar a consolidação de outras vertentes teóricas, como, por exemplo, da Escola de Copenhague,[8] em que a definição de segurança é ampliada para incorporar novas dimensões, como a econômica, a ambiental e a social (Buzan, Waever, De Wilde, 1998). Essa dinâmica se justifica na medida em que no último século fenômenos não militares, como as doenças infecciosas de ocorrência natural, continuam representando ameaças, sendo comum episódios endêmicos se tornarem epidêmicos ou pandêmicos.

Em um desses episódios, no fim de 2019, a Organização Mundial da Saúde foi notificada sobre a ocorrência de pneumonia em um grupo de pacientes na cidade de Wuhan, província de Hubei, na China. Denominado Sars-CoV-2, o vírus que causa a *Coronavirus disease 2019* (WHO, 2020) se disseminou por todos os continentes pela via expressa do fenômeno da globalização.

Na esteira da eclosão de doenças infecciosas, não é incomum observar processos de "decadência econômica, fragmentação social e desestabilização política" – gatilhos de tensões no sistema internacional que podem culminar em conflitos de diferentes formas (NIE, 2000). Ocorrências dessa natureza exercem pressão sobre o ambiente internacional e suscitam análises sobre segurança nacional, que, enquanto síntese de conservação e de desenvolvimento, conforme os princípios constitucionais, é o máximo valor jurídico tutelado pelo Estado. Um dos seus componentes principais – a defesa nacional, "assume um significado estritamente instrumental, e compreende todas as modalidades organizativas e funcionais destinadas a garantir os valores essenciais sintetizados no conceito de segurança" (Vergotinni, 1998, p.312).

Nos EUA, desde 2018, há uma estratégia nacional com o fim de "avaliar, prevenir, detectar, preparar, responder e se recuperar de ameaças biológicas" que, além do governo, envolve outros partícipes, como a indústria, a academia, entidades não governamentais e o setor privado (EUA, 2018, p.1).

Dentre as ações previstas, destacam-se a aceleração da pesquisa básica e aplicada e o desenvolvimento de vacinas, antibióticos, testes de diagnóstico etc., no sentido de prevenir

ARTIGO_Roberto_Rita_ACristina.indd 4 19/04/2024 14:12:08

ou reduzir a propagação de doenças infecciosas com potencial de risco para a segurança nacional, assim como a redução, o surgimento e a disseminação de micro-organismos patogênicos resistentes a antibióticos (EUA, 2018).

No caso dos antibióticos, essas ações se justificam diante de indicadores que demonstram haver, nas últimas décadas, uma incidência menor de pesquisa e desenvolvimento tecnológico, assim como de pedidos de patente depositados e patentes de invenção expedidas. As razões envolvem vários aspectos, como legislação, custos, dificuldades quanto à eficácia e segurança de novos produtos e aumento da resistência das bactérias (Katz et al., 2006).

Neste último aspecto, ainda que os antibióticos, obtidos por isolamento natural, por síntese química ou por derivação semissintética, tenham potencial para eliminar ou inibir a multiplicação de bactérias, não se pode deixar de considerar a capacidade de um micro-organismo sobreviver e até mesmo se replicar durante um tratamento, por meio da mudança de seu material genético, para se tornar insuscetível à substância (Mossialos et al., 2010). Afinal, não é só o homem que sofre alteração contínua em resposta a doenças infecciosas, os vários micro-organismos infecciosos que provocam doenças também passam por um processo de adaptação e ajustamento ao ambiente (McNeill, 1976, p.8-9).

Se a resistência aos antibióticos é observada como uma ameaça atual, um dos maiores desafios enfrentados, entre as duas grandes guerras, foi a ausência ou a escassez de substâncias eficazes para conter altos índices de mortalidade por doenças infecciosas causadas por bactérias. A mudança desse quadro decorreu das inovações nos setores químico e farmacêutico, da criação da Organização Mundial da Saúde (1948) e dos valores universais que, após a década de 1930, garantiram a extensão dos avanços na saúde (Acemoglu, Johnson, 2007, p.935-936).

Desses fatores, destaca-se o primeiro, em especial a trajetória tecnológica da penicilina, que, de acordo com um antigo editorial do *British Medical Journal* (Penicillin..., 1944), envolveu uma sequência de etapas: a descoberta, as pesquisas de como obter a substância relativamente pura, a demonstração de seu uso clínico e a produção em larga escala.

## Análise da trajetória tecnológica da penicilina

Apesar dos esforços ao longo da história na utilização de fungos para o tratamento de infecções bacterianas (Wainwright, 1989), coube a Alexander Fleming (1929), no curso das suas pesquisas nos laboratórios do St. Mary's Hospital, em Londres, em 1928, a descoberta[9] da ação antibacteriana de um fungo do gênero *Penicillium* em algumas culturas de bactérias, concluindo sobre a possibilidade do uso terapêutico.

No início do século XX não havia nenhuma política estabelecida para a ciência na Inglaterra, apenas respostas para contingências (Bud, 2018). Na condição de uma das principais autoridades em antissépticos, Fleming era qualificado para realizar uma avaliação da realidade (Sneader, 2005) e, talvez, assumir riscos e aceitar desafios para agregar valor à sua descoberta, resultando em invenção, mudança tecnológica ou introdução no mercado de um produto ou processo. Não foi, no entanto, o que ocorreu, uma vez que suas ações se revelaram mais em nível acadêmico e experimental do que em um ambiente empreendedor.

ARTIGO_Roberto_Rita_ACristina.indd 5 19/04/2024 14:12:08

Para Maclaurin (1953, p.100), contemporâneo de Fleming, a ciência básica nem sempre era uma via expressa para uma patente de invenção ou mudança tecnológica imediata, pois nem sempre o interesse do cientista consistia na exploração comercial de suas descobertas, geralmente conquistadas pela sua diligência vocacional e "impulso intelectual criativo de ampliar a compreensão dos fenômenos naturais".

Além disso, há outras questões que podem ser consideradas, como a possibilidade de que Fleming, diante das "tentativas fracassadas de isolar e extrair a penicilina", tenha decidido que "não valeria a pena enfrentar as dificuldades, os obstáculos imprevisíveis e incertezas, especialmente porque era bastante questionável se a produção seria economicamente viável" (Wolf, 1996, p.8), ou, ainda, diante de poucas pesquisas adicionais sobre o potencial clínico da penicilina além da aplicação ocasional para tratar infecções (Sneader, 2005), o fato de ter percebido "a parte menos importante do seu potencial" (Kingston, 2000, p.686), não conseguindo "entender a necessidade da estreita relação entre aqueles que observam fenômenos microbiológicos e eventos clínicos e aqueles que desenvolvem medicamentos" (Hobby, 1985, p.57).

Como uma criação pode resultar diretamente de novas descobertas científicas e o progresso técnico-científico pode envolver tanto a descoberta quanto a invenção e a inovação, a descontinuidade das pesquisas de Fleming como fenômeno estrutural pode ser entendida com a compreensão da natureza desses processos, que não são automáticos, e por isso devem ser diferenciados. Descoberta não é sinônimo de invenção, assim como invenção não é de inovação e nem todas as invenções e inovações são patenteadas. Existem requisitos que diferenciam inovação de outros conceitos, como invenção. Esta, embora seja resultado de atividades inventivas e esforços inovativos, com atividades de desenvolvimento em potencial para resultar em uma inovação, assim será definida quando em uso ou disponível no mercado. Há, portanto, para Schumpeter (1997), quanto ao processo de mudança tecnológica, a existência de três processos correlatos: invenção, inovação e difusão.

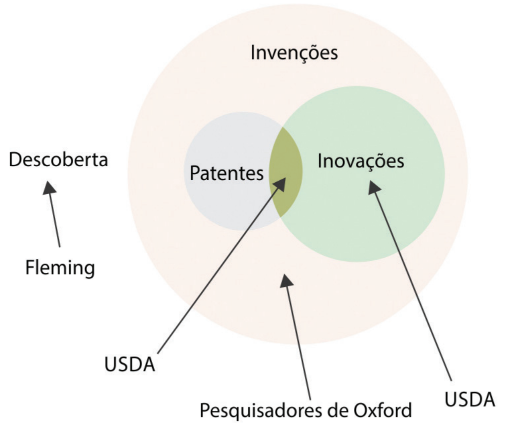

Figura 1: Aspectos da trajetória tecnológica da penicilina (adaptado pelos autores de Basberg, 1987)

ARTIGO_Roberto_Rita_ACristina.indd 6 19/04/2024 14:12:08

A Figura 1 sintetiza a trajetória tecnológica da penicilina, desde o acaso e a observação de Fleming, passando pela contribuição intelectual dos pesquisadores da Universidade de Oxford até chegar ao momento da pesquisa e desenvolvimento tecnológico do processo de produção em larga escala pelo United States Department of Agriculture (Usda, Departamento de Agricultura dos EUA). Os tamanhos do conjunto e dos subconjuntos foram definidos aleatoriamente, sem levar em consideração aspectos quantitativos. No conjunto das invenções, encontram-se os subconjuntos das invenções patenteadas e das invenções que se tornaram inovações, assim como a respectiva interseção entre elas – a menor parcela na relação estabelecida.

Na linha propugnada por Maclaurin (1953), observa-se que o estudo de caso da penicilina é didático, por oferecer fatores que são passíveis de mensuração no âmbito do processo de avanço tecnológico, como a predisposição para desenvolver ciência básica, inventar, inovar, apoiar e aceitar a inovação.

Após a Segunda Guerra Mundial, já havia a percepção de que o crescimento econômico era mais imediatamente afetado pela propensão a inventar e inovar do que pela propensão de desenvolver ciência básica, embora esta também fosse considerada essencial para o progresso tecnológico (Bush, 1945). Uma característica significativa da invenção, uma nova solução para um problema técnico específico (Ompi, 1978, p.5), é que ela "revela um método operacional de criação de algo novo" (Maclaurin, 1953, p.102); enquanto a inovação corresponde ao momento em que uma "invenção é introduzida comercialmente como um produto ou processo (p.105).

Além disso, a invenção de um produto ou processo novo e útil é uma coisa; a produção e o uso são outra coisa. Por isso não é fácil prever ou planejar o avanço tecnológico. A invenção requer inovação antes que ocorram mudanças econômicas, e o desafio para financiar ou realizar a produção e a frustração para comercializar novos produtos ou processos são de conhecimento comum (Nelson, 1959). Assim, para que "a inovação tecnológica aconteça, é necessário existir uma política específica que contemple a atividade, pois se trata de procedimentos, processos, parâmetros específicos, diferentes daqueles envolvidos propriamente na pesquisa científica" (Homma et al., 2003, p.672).

No caso da penicilina, todos esses processos devem ser destacados, pois dez anos após a descoberta de Fleming, momento em que o tratamento de doenças infecciosas bacterianas ainda se concentrava quase exclusivamente nas sulfonamidas e derivados[10] (Walsh, 1984), havia pouca atenção da comunidade científica e escassez de citações do respectivo artigo científico (Wolf, 1996, p.7). Com a revisão da literatura, a possibilidade relacionada a uma substância natural como a penicilina despertou o interesse científico de Howard Florey e Ernst Chain, ambos pesquisadores da Universidade de Oxford que, na tentativa de vencerem as resistências que se opunham à invenção e à inovação, em especial de pesquisa e produção, conseguiram o apoio financeiro da Rockefeller Foundation. Os resultados dos testes clínicos realizados com animais, obtidos com penicilina parcialmente purificada, demonstraram a eficácia da substância contra alguns organismos como estafilococos, estreptococos, pneumococos, assim como a ausência de toxicidade (Chain et al., 1940).

Posteriormente, com o desenvolvimento da pesquisa até o estágio da terapia humana, e a utilização de uma quantidade de penicilina parcialmente purificada, foram obtidas

ARTIGO_Roberto_Rita_ACristina.indd 7 19/04/2024 14:12:08

informações mais precisas da sua ação sobre as espécies de bactérias listadas. Se a penicilina foi descoberta por Fleming ao observar a habilidade de determinado fungo de inibir o crescimento de micro-organismos patogênicos, um dos maiores desafios para os pesquisadores de Oxford no curso das suas pesquisas se relacionava à invenção de um processo de produção em quantidade suficiente para uso terapêutico (Abraham et al., 1941).

Mesmo após a publicação dos resultados das propriedades da penicilina parcialmente purificada (Chain et al., 1940; Abraham et al., 1941), as atividades prosseguiram, sendo desenvolvido um processo para obter uma preparação de penicilina altamente purificada, cujos resultados faziam menção à penicilina como o agente antibacteriano "mais poderoso" até então conhecido (Abraham, Chain, Holiday, 1942).

Não se tinha ideia se a descoberta de Fleming, ainda pendente da invenção de um processo de produção em larga escala para alcançar a condição de inovação, poderia perturbar ou ameaçar a ordem ou os interesses estabelecidos no mercado dominado pelas sulfonamidas. Em que pese a publicação de dados e informações em periódicos pelos pesquisadores de Oxford, a guerra impediu que os alemães obtivessem os artigos pelas vias regulares, mas, ainda que com atraso, eles se interessaram na avaliação do valor científico e estratégico da penicilina; no entanto, as suas conquistas e progressos não podem ser avaliados ou medidos em separado da evolução anglo-americana (Horack et al., 1945; Shama, Reinarz, 2002).

Considerando que "novos produtos e novos processos não aparecem totalmente desenvolvidos, eles são baseados em novos princípios e novas concepções, que, por sua vez, são cuidadosamente desenvolvidos por pesquisas nos domínios mais puros da ciência" (Bush, 1945, p.17), a pesquisa básica que levou Fleming à descoberta da penicilina foi posteriormente complementada com a pesquisa aplicada na Universidade de Oxford, em especial sobre como obter a substância relativamente pura e a demonstração do seu uso clínico. A limitação do processo de produção inglês, porém, era um obstáculo para o alcance do potencial terapêutico da penicilina que ainda o separava de uma inovação radical (Stewart, 1948).

Portanto, se a "história de uma tecnologia é contextual à história das estruturas industriais associadas a essa tecnologia" (Dosi, 1982, p.147), assim como o "processo inovador, tanto em seus procedimentos 'normais' quanto em seus avanços 'extraordinários', é moldado pela interação de fatores econômicos e institucionais" (p.160), faz-se necessário destacar o protagonismo do Usda, cuja origem remonta a 1836, quando uma nova Lei de Patentes (EUA, 1836) estabeleceu e vinculou ao Departamento de Estado norte-americano um escritório denominado United States Patent Office (Uspo, Escritório de Patentes dos EUA).[11] Seu primeiro mandatário, o comissário Henry Ellsworth, por iniciativa própria, tornou a agricultura uma importante política governamental, com o desenvolvimento de programas de pesquisas e a publicação de estatísticas, o que motivou o Congresso norte-americano, em 1839, a autorizar a primeira despesa federal para o setor no valor de mil dólares. Quando já existia uma divisão agrícola no âmbito do Escritório de Patentes, seu pessoal assim como a biblioteca foram deslocados para o Departamento de Agricultura que o Congresso decidiu criar (EUA, 1862), com a competência de produzir e difundir informações úteis sobre assuntos relacionados ao setor. Em que pese o desmembramento, o Usda e o Uspo mantiveram-se em estreita cooperação (EUA, 1961).

ARTIGO_Roberto_Rita_ACristina.indd 8 19/04/2024 14:12:08

A propensão para apoiar inovações foi mais um elemento primordial observado por Maclaurin (1953), que envolvia também a oferta de capital para novos desenvolvimentos. Na Inglaterra, no período de 1920 a 1930, ocorreram contribuições relevantes para a ciência básica e aplicada, como, por exemplo, para o desenvolvimento do radar e motores a jato no início da Segunda Guerra Mundial. No caso da penicilina, conforme já observado, verifica-se o apoio financeiro da Fundação Rockefeller para os estágios iniciais dos estudos dos pesquisadores da Universidade de Oxford e o investimento do governo norte-americano, por meio do Usda, para o desenvolvimento do processo de produção em larga escala. Em linha semelhante, observam Brown, Cueto e Fee (2006, p.627) que "a Fundação Rockefeller, especialmente sua International Health Division (Divisão Internacional de Saúde), criada em meados da década de 1910, também foi um importante ator na saúde internacional no início do século XX".

A eclosão da Segunda Guerra Mundial alterou de forma considerável o cenário político e estratégico dos EUA. Até o seu início, os programas de pesquisa e desenvolvimento do governo norte-americano se consubstanciavam em um modesto esforço interno, principalmente no setor da agricultura, o que mudou depois com as necessidades da guerra e a implementação de ações relacionadas à investigação científica em vários segmentos (EUA, 1947), um dos motivos pelo qual foi criado o Office of Scientific Research and Development (OSRD, Escritório de Pesquisa e Desenvolvimento Científico) em 1941. A sua criação visava assegurar a provisão adequada para pesquisas sobre problemas científicos relativos à defesa nacional que, em termos de atividades de pesquisa e desenvolvimento tecnológico, ficava apenas atrás do Departamento de Guerra (EUA, 1941, 1947).

Assim, levando em conta que os pesquisadores de Oxford demonstraram a eficácia antibacteriana da penicilina, mas conseguiram produzir apenas pequenas quantidades, e a indústria britânica não estava determinada e não detinha competência para elevar a produção (Bud, 2011), o Usda, com o seu histórico de fermentação de fungos, foi instado a auxiliar o respectivo programa de pesquisa e desenvolvimento tecnológico. Sob o custo de cem mil dólares em salários e despesas do governo norte-americano, a maioria das vias usadas para a produção de penicilina utilizava o cultivo do organismo por meio de cultura de superfície, o que, para a produção em larga escala, tornava-o um processo difícil e caro. Esse cenário se modificou com a obtenção de linhagens de maior rendimento, purificação da substância e o desenvolvimento de processos, um deles relativo à produção por meio de cultura submersa – econômico e viável para fins terapêuticos, que se manteve como o principal até a síntese química da penicilina (EUA, 1961).

O programa de pesquisa e desenvolvimento tecnológico implementado pelo governo norte-americano sobre a estrutura e síntese da penicilina durou cerca de dois anos e envolveu laboratórios, universidades, profissionais e agências governamentais, assim como um acordo com o Reino Unido para a participação de empresas e agências britânicas. Todos os envolvidos concordaram em compartilhar informações e permitir visitas às suas instalações. Nesse caso, o compartilhamento mútuo dos resultados das pesquisas foi realizado pelo próprio OSRD, e, no que concerne aos pedidos de patente, em todos os casos, os EUA tinham a garantia quanto ao uso governamental (EUA, 1947), conforme será possível observar adiante.

ARTIGO_Roberto_Rita_ACristina.indd 9 19/04/2024 14:12:08

A propensão para aceitar inovações varia entre grupos e regiões, mas durante a Segunda Guerra Mundial, nos EUA, esse era um processo rápido e vinculado à capacidade da inovação de atender a uma necessidade real e o produto ou processo avançar ao estágio em que os principais obstáculos iniciais fossem superados (Maclaurin, 1953). No caso da penicilina, ao longo da década de 1930, o que foi produzido e utilizado se resumia à escala de laboratório e era quase exclusivamente para fins experimentais. Com as atividades de pesquisa e desenvolvimento tecnológico de um processo de produção em larga escala houve a disponibilidade do produto, em grandes quantidades, para uso civil e militar. Porém, antes de atingir esse patamar em junho de 1945, com a produção mensal de seiscentos milhões de unidades, o quadro era diametralmente oposto. Em 1941 e 1942 foram necessários 18 meses para produzir penicilina suficiente para o tratamento de duzentas pessoas (Stewart, 1948). Na prática, esse avanço foi responsável, em parte, pela diminuição dos índices de mortalidade de várias doenças no Exército americano para cada mil soldados, com os resultados demonstrando que a taxa caiu de 14,1 para 0,6 no período entreguerras (Bush, 1945).

## Resultados na área de propriedade intelectual

Compreendida como um conjunto de direitos exclusivos temporários outorgados pelo Estado ao titular de uma invenção nova, não óbvia e industrialmente aplicável, a patente é uma das formas mais antigas de proteção à propriedade intelectual e, possivelmente, a de maior relevo para a defesa nacional, por oferecer mais possibilidade de inovação. Daí a importância do sistema de patentes – "um conjunto de normas e princípios jurídicos considerado em sua perspectiva socioeconômica" (Carvalho, 1982, p.224) – como um sistema dinâmico de controle e monitoramento de tecnologias estratégicas.

Para o enquadramento como tecnologia estratégica, é necessário que uma invenção (produto ou processo), modelo de utilidade, assim como eventual certificado de adição, revelem um fator de risco para a segurança nacional em caso de publicação; e/ou potencial para atender às demandas de defesa nacional (Silva, 2016, 2022; Silva, Pinheiro-Machado, 2022). Ainda que, em um ordenamento político, o conceito de defesa seja regularmente "associado à sua organização militar" (Vergotinni, 1998, p.312), tal expressão "não significa apenas a organização da força armada" (Penna, 1918, p.19). Então, independentemente da origem e aplicação do pedido de patente (civil ou militar), em ambos os casos o seu objeto pode ser assim enquadrado.

*Mutatis mutandis*, ao programa de pesquisa e desenvolvimento tecnológico do processo de produção em larga escala de penicilina foi aplicada a mesma filosofia do programa de pesquisa e desenvolvimento relativo aos artefatos nucleares no âmbito do Manhattan Project (Bush, 1945; Wellerstein, 2008). Na prática, isso significa que a coordenação e o apoio do governo norte-americano, via OSRD, assim como os esforços de guerra, foram determinantes para que a produção em larga escala de penicilina tenha sido implementada no momento oportuno.

Houve, portanto, por certo, a figura do empresário inovador apresentada por Schumpeter (1997), representada pelo papel do Estado como o agente econômico fundamental que trouxe à prática do mercado um processo que possibilitou a produção em larga escala de penicilina. Desse modo, ao examinar a sequência ciência-tecnologia-produção relativa à

ARTIGO_Roberto_Rita_ACristina.indd 10 19/04/2024 14:12:08

penicilina, cabe destacar a pesquisa e o desenvolvimento de novos e eficientes meios de produção no ambiente de inovação do Usda.

Daí os resultados na área de propriedade intelectual, que no período de 1943 a 1945 foram apresentados ao Uspo sob a forma de pedidos de patente, por iniciativa do Usda, do OSRD e dos próprios inventores (Best, Bradley, 2020; Gross, Sampat, 2021), no total de oito requerimentos.[12] Os dois primeiros tinham como objeto invenções de processos para o isolamento da penicilina, cuja estrutura química ainda não era conhecida naquele momento, ao contrário de suas propriedades bacteriostáticas e da sua falta de estabilidade quando submetida a diversas condições químicas ou físicas. Além disso, havia outras dificuldades como: a remoção incompleta de penicilina do meio biológico bruto em que ocorria; a destruição de penicilina durante o processamento; e a contaminação do concentrado final com impurezas. As duas primeiras dificuldades estavam associadas à perda de penicilina, tornando a produção inviável do ponto de vista econômico. A terceira conduzia o produto a uma condição impura que, além de apresentar um aspecto desagradável e reduzida potência biológica, poderia conter substâncias biologicamente ativas indesejáveis, limitando a sua aplicação terapêutica. Os procedimentos até então utilizados para isolamento, purificação e concentração de penicilina não superavam essas dificuldades e, ademais, exigiam o uso oneroso de produtos químicos, equipamentos e tempo (Watchel, 7 maio 1946, 16 dez. 1947).

Assim, apesar dos avanços, de já classificada como um empreendimento comercial e das tentativas de variações nos constituintes usuais do meio nutriente e nas condições de operação, a produção de penicilina ainda apresentava baixo rendimento, motivando a invenção de um processo para aumentar o desempenho da substância, originando um novo pedido de patente em 1944 (Coghill, Moyer, 15 jul. 1947).

Ainda como uma questão pendente de solução, a equação da produção de penicilina em larga escala, suficiente para uso terapêutico, foi solucionada com três novas invenções, cujos processos deram origem a três novos pedidos de patente. Esses novos processos tinham valor não apenas devido ao alto rendimento de penicilina obtido, mas também porque possibilitavam considerável economia na operação do processo de fermentação (Moyer, 25 mar. 1948, 22 jun. 1948, 12 jul. 1949).

Em 1945, foram realizados os últimos depósitos de pedidos de patente, relacionados com as novas composições de matéria e, mais particularmente, com compostos da família da penicilina. Nos relatórios descritivos de dois pedidos de patente consta que uma das espécies, conhecida como *Parahydroxypenicillin G*, foi isolada pela primeira vez e identificada como um derivativo (Stodola, Watchel, Coghill, 6 nov. 1951, 7 maio 1950).

Paralelamente, foi aprovado pelo Congresso norte-americano o Science Mobilization Bill (EUA, 1943) – um plano para a máxima utilização e coordenação da ciência e tecnologia durante a Segunda Guerra Mundial. Suas disposições previam, entre outras operações, o estabelecimento de um escritório de mobilização científica e tecnológica, o financiamento do avanço da pesquisa pura e aplicada e o manejo de direitos de propriedade intelectual aplicáveis a descobertas e desenvolvimentos financiados pelos EUA, quando de interesse público. Nesse período, já vigia uma lei que outorgava ao secretário do Interior e ao comissário de Patentes autoridade para conceder a qualquer servidor do governo, exceto aos funcionários do próprio escritório, uma patente para qualquer invenção permitida por

ARTIGO_Roberto_Rita_ACristina.indd 11 19/04/2024 14:12:08

lei, quando tal invenção fosse usada no serviço público, sem o pagamento de qualquer taxa (EUA, 1883 citado em EUA, 2019, p.25).

Naquele contexto, os servidores de departamentos governamentais dos EUA, como resultado de suas funções, tinham amplas oportunidades para inventar e inovar, e a maioria das invenções resultantes dos gastos com pesquisas fluíam dos seus próprios esforços (EUA, 1961, p.6). Porém, em razão do baixo grau de efetividade da lei de 1883 em mais de 40 anos, sobretudo pela falta de observância pelos próprios destinatários dos procedimentos que regiam as relações estatutárias e contratuais, passou a ser exigida na alteração de 1928 a certificação pelo requerente no pedido de patente de que a invenção descrita e reivindicada, se patenteada, poderia ser fabricada e usada pelo ou para o governo dos EUA para fins governamentais sem o pagamento de qualquer remuneração[13] (EUA, 1883 citado em EUA, 2019, p.25; EUA, 1924, p.1; EUA, 1928 citado em Kreeger, 1947, p.720; EUA, 1960, p.6-10).

Assim, em que pesem a pesquisa e o desenvolvimento tecnológico do processo de produção em larga escala de penicilina se apresentarem como um exemplo contundente de que "a proteção patentária não era necessária para todo o fluxo das principais inovações farmacêuticas" (Dosi et al., 2021, p.3), cabe destacar que a questão política estratégica foi essencial para viabilização do empreendimento. Na prática, isso significa que, a partir do aperfeiçoamento da lei e a proteção patentária, os resultados de programas e projetos puderam ser mais bem manejados, visto que havia a garantia de que o governo dos EUA obteria direitos suficientes sobre as invenções de forma a atender ao interesse público, assim como minimizar os custos de políticas, principalmente para atender às demandas de defesa nacional.

Em todos os pedidos de patente relativos à penicilina, os EUA foram representados pelo secretário do Departamento de Agricultura e pelo diretor do OSRD, e o perfil dos resultados, em sua maioria, relacionou-se a processos, o que é justificável, por se apresentar naquele momento como um dos maiores desafios de inovação tecnológica.

Como cada ambiente social tem suas próprias formas de preencher a função empresarial (Schumpeter, 1997), coube ao governo americano, por meio da OSRD e Usda, atuar nesse sentido até o fim da Segunda Guerra Mundial, momento em que o setor industrial já estava estabelecido. Naquele contexto, o valor da penicilina já estava além de uma estimativa econômica, em face de sua imensa contribuição para a segurança nacional, e algumas das patentes relacionadas aos processos de produção de penicilina em larga escala já haviam sido licenciadas a laboratórios farmacêuticos[14] (EUA, 1961).

Por tudo isso, é possível afirmar que a descoberta da penicilina, os esforços intelectuais dos pesquisadores de Oxford, a ação estratégica do governo americano para o desenvolvimento de um processo de produção em larga escala e o novo ciclo dos antibióticos foram elementos-chave para impulsionar a indústria farmacêutica dos EUA e transformá-la em um dos setores estratégicos, o que permitiu a sua reorganização em torno de um novo modelo de negócio com base na inovação (Neushul, 1993), estabelecendo programas de pesquisa e desenvolvimento tecnológico e a introdução de novos medicamentos (Henderson, Orsenigo, Pisano, 1999, p.269). Dessa forma, o antibiótico se tornou um recurso político, além de médico, fomentando não apenas a replicação de processos e produtos em escala global, mas também novas pesquisas e desenvolvimentos nesse setor (Bud, 2011).

ARTIGO_Roberto_Rita_ACristina.indd 12 19/04/2024 14:12:09

## Considerações finais

A função estratégica das patentes de invenção, há muito tempo um componente relevante de políticas públicas, demandou de vários países, desde o início do século XX, o enfrentamento de escolhas complexas sobre como adaptar e utilizar as suas leis para melhor servir aos seus objetivos políticos e responder aos fatores dominantes, como, por exemplo, a mudança tecnológica e os seus impactos para a defesa nacional.

As demandas relativas a doenças infecciosas no período entreguerras, como, por exemplo, algumas de origem bacteriana, determinaram os grandes esforços do governo americano para mobilizar a ciência e implementar ações visando à pesquisa e ao desenvolvimento tecnológico de um processo de produção em larga escala de penicilina – um dos pontos focais na Segunda Guerra Mundial.

Em contraste com o *status quo ante* relativo à descoberta de Fleming e dos esforços intelectuais dos pesquisadores de Oxford, o modelo de inovação do OSRD, com forte ênfase na pesquisa aplicada, devido à urgência da guerra, induziu relações entre governo, universidades e empresas farmacêuticas capazes de transformar a penicilina em um produto de defesa.

Tais iniciativas do governo americano nesse período se refletiram inicialmente na defesa nacional, expandiram-se para atender às necessidades da saúde pública e repercutiram na economia – todos componentes da segurança nacional. Os resultados podem ser medidos pelos produtos e processos resultantes que, posteriormente, foram objeto de pedidos de patente perante o Escritório de Patentes dos EUA.

Principalmente quando aplicada a medicamentos e vacinas, a propriedade intelectual, em todas as suas modalidades, especialmente as patentes, é motivo de grande controvérsia. Como uma parte do avanço tecnológico em questão consistiu em inovações, as quais foram patenteadas, seu uso e aproveitamento pelo governo dos EUA sem a necessidade de remuneração foram garantidas por disposições legais específicas.

O governo americano foi um ator potencialmente importante naquele contexto, mas, em que pesem os resultados, a replicação na atual conjuntura – aventada recentemente por estudiosos e formuladores de políticas – da política adotada durante a Segunda Guerra Mundial, cuja experiência exitosa resultou no desenvolvimento de um processo de produção em larga escala de penicilina, não necessariamente significa unicidade de solução, dada a existência de variáveis referentes aos contextos social, histórico, econômico e político de origem.

O cenário político e econômico internacional presente é diverso do passado, sobretudo no aspecto da concentração do mercado global de fármacos. Conforme observado, no aspecto da propriedade intelectual, por exemplo, as patentes de invenção não se constituíram em um ponto vital no rápido desenvolvimento do processo de produção em larga escala de penicilina. Ressalvados possíveis aspectos políticos, legais e estruturais então limitantes na Inglaterra, Fleming e os pesquisadores de Oxford não depositaram nenhum pedido de patente relacionado aos resultados de suas pesquisas. Além disso, com base na sua política governamental, os EUA não permitiram que uma ou mais empresas do segmento farmacêutico naquele momento controlassem o mercado, tendo inclusive licenciado as tecnologias patenteadas após a guerra para vários interessados, sem a fixação

ARTIGO_Roberto_Rita_ACristina.indd 13 19/04/2024 14:12:09

de remuneração. A conjugação de esforços de inovação tecnológica para a produção de penicilina em várias frentes, visando atender principalmente à demanda da Segunda Guerra Mundial, permitiu a formação de uma cadeia de suprimentos estratégica de forma a evitar concentração e interrupções diante das necessidades existentes, incluído o fornecimento para o meio civil.

Nem sempre políticas públicas que foram eficazes no passado são passíveis de reaplicação no presente, sobretudo quando diante de um necessário e complexo envolvimento de governos, indústrias e instituições acadêmicas para o desenvolvimento de uma solução eficaz contra agentes biológicos que ameaçam em menor ou maior grau a segurança nacional de vários países em períodos distintos da história.

A certeza é que eventos biológicos, como, por exemplo, a covid-19, demonstram a indissociabilidade entre vários componentes da segurança nacional, porquanto não se deve negligenciar o seu potencial de causar mortes, provocar crises nos mercados, instabilidade social e insegurança global.

## NOTAS

[1] Nessa e nas demais citações em outros idiomas a tradução é livre.

[2] No século XVII, a "descoberta" dos micro-organismos foi relacionada ao microscópio desenvolvido por Anton van Leeuwenhoek (1632-1723). No século XIX, com as pesquisas de Emil Behring (1854-1917), Florence Nightingale (1820-1910), Ignaz Semmelweis (1818-1865), John Snow (1813-1858), Joseph Lister (1827-1912), Louis Pasteur (1822-1895), Jules Joubert (1834-1910), Paul Ehrlich (1854-1915) e Robert Koch (1843-1910), foi possível estabelecer definitivamente uma relação entre doenças infecciosas e micro-organismos patogênicos.

[3] Dosi (1982, p.148) denomina "trajetória tecnológica" a direção do avanço dentro de um paradigma tecnológico – um conjunto de procedimentos, uma definição dos problemas "relevantes" e dos conhecimentos específicos relacionados com a sua solução.

[4] Esses resultados evidenciam a distinção do olhar de Carl von Clausewitz (1780-1831) sobre o fenômeno da guerra, pois os seus manuscritos já destacavam dentre outros aspectos, as perdas flagrantemente desproporcionais de um exército derrotado, assim como de um exército vitorioso, com um número maior de baixas devido a doenças do que ao combate (Clausewitz, 1984, p.207, 270).

[5] No período entreguerras houve depósitos de pedidos de patente de novos compostos com ação contra os protozoários causadores da malária. Ver patente US 2,233,970. Disponível em: https://patentimages.storage.googleapis.com/09/71/81/e831fd5901b121/US2233970.pdf. Acesso em: 10 out. 2020.

[6] Há controvérsias se o termo é de autoria de Selman Waksman em 1942, por ocasião da descoberta da estreptomicina, ou de Paul Vuillemin, que introduziu o conceito de "antibiose" em 1889, processo no qual um organismo produz uma substância que impede ou dificulta o crescimento de outro organismo (Morales, Caldas, 2010, p.14).

[7] O *Atlas of diseases* (1954) foi apoiado na época pelo Escritório de Pesquisa Naval da Marinha dos EUA. Disponível em: https://collections.lib.uwm.edu/digital/collection/agdm/id/5697. Acesso em: 10 mar. 2021.

[8] "Emprega uma abordagem crítica aos estudos de segurança", o que envolve, dentre outros tópicos, a análise "das ameaças aos Estados (ou seja, a segurança nacional) como questões que são socialmente construídas" (Romaniuk, 2018, p.2).

[9] Pode ser definida como o ato de "obter, pela primeira vez, a visão ou o conhecimento de algum fato ou princípio até então desconhecido" (Bush, 1945, p.61), assim como "o reconhecimento de fenômenos, propriedades ou leis do universo material não reconhecidos até então e passíveis de verificação" (Ompi, 1978, p.5).

[10] Paul Gelmo (1879-1961), com um método de síntese da sulfanilamida em 1908; Heinrich Hörlein (1882-1954), com a patente DE 226,329 em 1909; Gerhard Domagk (1895-1964), com os resultados de suas pesquisas sobre sulfonamida em 1932; e o conglomerado IG Farben Bayer, com a introdução no mercado em 1935 do Prontosil Rubrum (sulfonamida).

ARTIGO_Roberto_Rita_ACristina.indd 14 19/04/2024 14:12:09

[11] Em 1975, com o Patent Office Name Change Act (88 Stat. 1949), o United States Patent Office (Uspo) passou a ser denominado United States Patent and Trademark Office (Uspto).

[12] Patentes US 2,399,840, US 2,423,873, US 2,432,638, US 2,442,141, US 2,443,989, US 2,476,107, US 2,504,161 e US 2,573,741.

[13] Para uma melhor compreensão do cumprimento do texto da lei de 3 de março de 1883, alterada pela lei de 30 de abril de 1928, é indicado observar as declarações realizadas pelos requerentes no primeiro parágrafo dos textos dos pedidos de patente relacionados à penicilina (ver nota 12 e referências).

[14] US 2,423,873 (Schenley Laboratories, 1947; Merck, 1948; Edward Robinson Squibb and Sons, 1948); US 2,442,141 (Eli Lilly and Company, 1948; Edward Robinson Squibb and Sons, 1951; Schenley Laboratories, 1949); US 2,443,989 (Eli Lilly and Company, 1948; Edward Robinson Squibb and Sons, 1948; Abbott Laboratories, 1949; Merck, 1949); e US 2,476,107 (Eli Lilly and Company, 1949; Edward Robinson Squibb and Sons, 1951; Schenley Laboratories, 1949; Merck, 1949).
